# Open innovation and external sources of innovation. An opportunity to fuel the R&D pipeline and enhance decision making?

**DOI:** 10.1186/s12967-018-1499-2

**Published:** 2018-05-08

**Authors:** Alexander Schuhmacher, Oliver Gassmann, Nigel McCracken, Markus Hinder

**Affiliations:** 1 Reutlingen University, Alteburgstrasse 150, 72762 Reutlingen, Germany; 2 Institute for Technology Management, University of St. Gallen, Dufourstrasse 40a, 9000 St. Gallen, Switzerland; 3 Debiopharm International S.A., Chemin Messidor 5-7, 1002 Lausanne, Switzerland; 4 Novartis Institutes for BioMedical Research, Postfach, Forum 1, 4002 Basel, Switzerland

**Keywords:** Open innovation, Pharmaceutical industry, Public–private partnerships, Crowdsourcing, Knowledge leverager

## Abstract

Historically, research and development (R&D) in the pharmaceutical sector has predominantly been an in-house activity. To enable investments for game changing late-stage assets and to enable better and less costly go/no-go decisions, most companies have employed a fail early paradigm through the implementation of clinical proof-of-concept organizations. To fuel their pipelines, some pioneers started to complement their internal R&D efforts through collaborations as early as the 1990s. In recent years, multiple extrinsic and intrinsic factors induced an opening for external sources of innovation and resulted in new models for open innovation, such as open sourcing, crowdsourcing, public–private partnerships, innovations centres, and the virtualization of R&D. Three factors seem to determine the breadth and depth regarding how companies approach external innovation: (1) the company’s legacy, (2) the company’s willingness and ability to take risks and (3) the company’s need to control IP and competitors. In addition, these factors often constitute the major hurdles to effectively leveraging external opportunities and assets. Conscious and differential choices of the R&D and business models for different companies and different divisions in the same company seem to best allow a company to fully exploit the potential of both internal and external innovations.

## Background

Traditionally, pharma R&D organizations are fully integrated; they are composed of various units with different competencies, skills and technologies and have complex interfaces to accomplish the multi-disciplinary tasks required for developing new drugs. Regulatory hurdles, complex research for new drug targets, or the low predictability of animal models are some examples of why the industry is struggling with its R&D input/output-ratio [1, 2]. These internal and external challenges made it necessary for companies to improve their R&D efficiencies, e.g. by outsourcing to reduce overhead costs, by the installation of proof-of-concept (PoC) organizations or by enhanced scientific rigor in data-driven project decision-making. The latter is included today in many (if not most) decisions that take into account the probability of technical success (PoS), the likelihood of achieving the target product profile (TPP) and the resources needed to achieve the TPP. The translation of innovations from research into clinically meaningful therapies for patients has become the key value generating step in pharmaceutical R&D. Nonetheless, in view of the high complexity of clinical PoC, the attrition rate in the PoC phase is still the highest. This raises the question regarding whether a decentralized and more open R&D organization that accesses additional competencies, skills and technologies from external sources could be a measure to increase the R&D efficiency.

Generally, a centralized R&D approach is particularly favoured and relevant in disease areas that necessitate large and long clinical trials to demonstrate PoC and where deep developmental, regulatory and commercial expertise is essential. Whenever an R&D activity is more singular and autonomous or as soon as less synergy within a portfolio of R&D projects exist, a decentralized R&D may have advantages over a central R&D organization since the company has the opportunity to copy the science-based biotech business model. Hence, pharma companies have increasingly moved away from internal R&D models towards more open and collaborative R&D models following the paradigm of open innovation [3]. In this vein, they have established specific collaborations with academic centres of excellence, built innovation centres, made joint ventures with academic institutions (public–private partnerships, PPP), established precompetitive consortia, or experimented with crowdsourcing and virtual R&D [4–7]. Some models even let competitors collaborate and become partners [8]. Currently, many companies have put greater focus on leveraging external knowledge, licensing or acquiring drug candidates and changing their R&D models from primarily inside-driven concepts to plans that more closely follow the open innovation paradigm [4]. As a consequence, the proportion of externally sourced R&D assets has increased in the past years [9]. This rise in externally sourced innovation coincided with several cases of major downsizing in R&D departments, with Merck, AstraZeneca and Pfizer being the biggest proponents of the R&D cuts [10–14].

This paper summarizes the current situation of open innovation in the pharmaceutical sector. It describes the concepts and models of opening up pharma R&D, and it attempts to address the questions regarding why big pharma has not yet fully embraced the different ideas of open innovation and what measures would help to increase R&D productivity and efficiency.

## Open source and crowdsourcing to access knowledge of the masses

Open source innovation has been successfully applied in the software industry. This radical open model of software development allows anyone to participate, network in the product’s development and then share the results. Rewards for participants are mostly non-monetary but are related to recognition and participation, such as the presentation of expertise, the satisfaction by working on an honourable project, the acquisition of new skills, or peer recognition. Around the time when internet became a commodity, the players in the pharmaceutical sector became more connected, and in turn, it was possible for R&D organizations to become more decentralized. This development allowed pharmaceutical companies to more easily access knowledge and ideas from the outside, promote innovation, use the mass of external experts to solve problems and to develop technologies through collaborative improvements [15, 16]. For example, the Human Genome Project has been a pioneering open source activity in the biomedical environment [17]. In addition, open-access scientific journals follow the principle of the free distribution of scientific information and knowledge. Other examples that are known are from non-profit organizations and governments to discover drugs for neglected diseases in collaboration with pharma companies [18]:

- In 1974, the Special Program for Research and Training in Tropical Diseases (TDR, http://www.who.int/tdr/en/) was initiated by the World Health Organization (WHO) as a global programme of scientific collaboration to combat neglected diseases [18]. The programme is hosted at the WHO and funded by co-sponsors, governments, foundations and agencies, such as the World Bank, the European Commission, or the Bill and Melinda Gates Foundation [19]. The African Network for Drugs and Diagnostics Innovation (ANDI) is a programme of the TDR that started in 2008 under the governance of the United Nations Economic Commission for Africa (UNECA). It focuses on the specific health needs in Africa and aims at promoting and supporting pharmaceutical R&D for neglected diseases led by African institutions to develop capacities and centres of research excellence there [20].
- The Medicines for Malaria Venture (MMV, https://www.mmv.org) was established in 1999 by a few European governments together with the World Bank to reduce the disease burden of Malaria infections [21]. Since then, the programme has gained more facilitators and received total funding of roughly USD 1 billion by 2016. The main sponsor is the Bill and Melinda Gates Foundation, with a 54.7% share. Partners form the industry include Novartis, Sanofi, Merck Serono and Takeda. The achievements are enormous. Several hundred million treatments were provided to children; 18,000 healthcare workers were trained to administer malaria medication; and 19 drug candidates have been nominated for clinical development since 1999. Since 2010, the MMV moved 17 candidates into preclinical development, of which 13 are still active [74].
- The Global Alliance for Tuberculosis Drug Development (TB Alliance, https://www.tballiance.org) is an initiative of governmental and non-governmental organizations. It was started in 2000 to discover and develop tuberculosis drugs. Today, the TB Alliance manages the largest pipeline of new TB drugs, including six new products that are in clinical development phases. Several pharmaceutical companies, such as Sanofi, Bayer or GSK have entered into collaborations with TB alliance in the past, whereas more recently Novartis transferred an entire R&D program to the TB alliance [71].
- A further open source example is the Drugs for Neglected Diseases Initiative (DNDi, https://www.dndi.org) that is supported and sponsored by numerous universities, research centres, governmental organizations, biotech companies, and several pharmaceutical companies, such as AstraZeneca, Bayer Healthcare, Bristol-Meyers Squibb (BMS), Genomics Institute of the Novartis Research Foundation, GSK, Pfizer, Sanofi and Takeda. It provides platforms for collaborative non-for-profit drug discoveries and developments for diseases such as Leishmaniasis, Sleeping Sickness, Chagas disease and paediatric HIV. Since 2003, DNDi developed four new drugs for the treatment of neglected diseases and plans to deliver 16-18 total new treatments with a total budget of EUR 650 million by 2023.
- Open source drug discovery (OSDD) was initiated by the Indian government in 2008 as a translational platform for drug discovery [22]. The hybrid model of open source and public private partnership brought together companies, organizations and scientists from different industries (such as TCG Lifesciences, Sun Microsystems, the Institute of Genomics and the Integrative Biology or Sky Quest Labs) in order to establish high-quality research on neglected diseases (such as tuberculosis) at low costs [69]. In its Connect to Decode initiative, the 4000 genes of *Mycobacterium tuberculosis* were annotated, and a metabolome and a protein–protein functional network of this germ were delivered [69]. OSDD also provides data on promising exploratory tuberculosis drugs via its website (http://www.osdd.net/home) and supports the progress of drug discovery projects [70].
- Finally, the Pool for Open Innovation started in 2009 as a partnership of GSK, Alnylam Pharmaceuticals and the Massachusetts Institute of Technology (MIT). It provides free access to 2300 tropical disease patents and ensures that these intellectual property (IP) rights do not hinder drug discoveries on neglected diseases.

These examples show how open sourcing in the pharma sector is being used to make drug development for neglected diseases feasible, thus ensuring affordable drugs for those who need them [18]. In addition, it reflects the important social function that the pharmaceutical industry has to play in developing new medications in financially unattractive markets. However, this does not perfectly fit with the pharmaceutical business model, which is built on large R&D investments, confidentiality, proprietary IP and returns-on-investment, and does not serve the needs of the industry to increase productivity in its core businesses [23].

Crowdsourcing is “*a form of sourcing in which individuals or organizations solicit contributions from Internet users to obtain desired services or ideas*” and has been described as a new business practice [24, 25]. Like open sourcing, crowdsourcing uses the mass of worldwide experts to get problems solved. Companies act as seekers and brokers. They post their questions or issues to a large, company-unknown, diverse group of experts and invite them to provide solutions. The external experts who are the solvers tackle the problems in return for financial gratification. Mining crowd data, web search logs, smart phone applications, social media, and active crowdsourcing are types of crowdsourcing that have found their way into the pharmaceutical business [26].

Active crowdsourcing is a cost-efficient way of open innovation that goes far beyond the more traditional models of research collaborations and partnerships [27–30]. The advantages include:

- The integration of external knowledge,
- The access to new technologies,
- The solving of problems that could not have been solved by internal experts, and
- A reduction of overhead costs.

The formal hurdles to implement successful active crowdsourcing are relatively low. The initiative needs to be publicized properly, the submission process needs to be simple and non-bureaucratic, and questions and challenges need to be precisely defined [31]. Hence, it is of great surprise that most of the big pharmaceutical companies apply only a seeker-like approach to get single problems solved by collaborating with platform providers (such as Innocentive) rather than hedging the full potential of crowdsourcing (Table 1).

**Table 1:** Examples of crowdsourcing in the pharma industry

Type of crowdsourcing	Example	Owner	Topics
Mining crowd data	Open FDA	FDA	Access to publicly available FDA data
Web search logs	Google trends	Google	Statistical analyses on health topics
Smart phone applications	Flumoji	GSK	Track flu outbreaks
Active crowdsourcing (pharma-specific platform)	Grants4Apps	Bayer	Electronic health
	Grants4Leads	Bayer	Drug discovery in animal health
	Grants4Targets	Bayer	Target proposals
	Open innovation drug discovery	Eli Lilly	Drug discovery Molecular modelling In vitro screening Drug discovery in animal health Compound proposals and synthesis NTD research
	Open innovation platform	AstraZeneca	Drug discovery Target proposals Data mining Chemoinformatics Preclinical and clinical translational research

So far, only Eli Lilly, Bayer Healthcare and AstraZeneca run their own crowdsourcing platforms:

- Eli Lilly left an early mark with Innocentive (2001), YourEncore (2003), and Open Innovation Drug Discovery (https://openinnovation.lilly.com/dd/) [32].
- AstraZeneca runs an open innovation platform (https://openinnovation.astrazeneca.com) that provides access to innovative discovery technologies.
- And Bayer HealthCare initiated Grants4Targets (G4T) (2009) [33, 34], Grants4Leads (2014; http://www.grants4leads.com), and Grants4Apps (2013, https://www.grants4apps.com). G4T is an open innovation platform to access new drug discovery ideas to fill Bayer Healthcare’s R&D pipeline. The process is simple and fast. External scientists are invited twice a year to propose their ideas for targets and an animal model. The administrative hurdles are low since the IP stays with the inventor. The proposals are evaluated by Bayer scientists, and if a proposal is accepted, the proposer receives a financial reward. If a proposal results in a project that finds its way officially into Bayer’s drug pipeline, then both parties can negotiate a collaborative agreement. So far, G4T has been very successful, as indicated by more than 1100 applications and inputs into 10 drug discovery projects that have led to 6 lead generations, 1 lead optimization and 2 drug development projects [34].

Other examples regarding how crowdsourcing is used by pharmaceutical companies are as follows:

- Novartis partnered with PatientsLikeMe (http://www.patientslikeme.com) [35], a digital health learning system comprising more than 600,000 participants’ health conditions.
- Novartis also uses the digital trial technology of Science 37 to allow patients an easy participation in clinical trials and to move more and more to a site-less trial model.
- A recent new partnership of Novartis is the one with Pear Therapeutics on a digital therapeutic called THRIVE [72].
- Merck & Co. collaborated with Kaggle (http://www.kaggle.com) [36] to identify statistical techniques for predicting the biological activities of different molecules.
- Sanofi has applied a more focused approach around Diabetes using the Data Design Diabetes Innovation Challenge to seek service solutions for diabetes patients.
- GSK uses crowd sourced data and its Flumoji app to identify the fluctuations of flu infections [37].
- And BioMedX (https://bio.mx/) is an example of a new open innovation model at the interface of crowdsourcing and innovation centres that includes pharma companies, such AbbVie, Boehringer Ingelheim, Johnson & Johnson (J&J) or Merck and academia. Physically based on the campus of Heidelberg University, experts from universities and pharma partners support the interdisciplinary projects of teams of young scientists. Anyone (the crowd of scientists) can apply via the internet and can ask for financial and scientific support to study new drug discovery ideas in the biomedical field (oncology, respiratory diseases, neuroscience, diagnostics and consumer care) with the purpose of translating the research activities into drug development projects of the respective pharma partner.

### Public private partnerships to improve competitiveness

PPPs have emerged as another cost-efficient way to propel drug innovations [38, 39]. These business ventures are financed and operated by a partnership of academic or publicly funded institutions, charities [such as the Bill & Melinda Gates Foundation (http://www.gatesfoundation.org)], and pharmaceutical companies. PPPs contribute to drug discovery efforts and allocate their resources primarily in niche indications with high unmet medical needs in which pharma companies are traditionally less active. In these open networks, the participants exchange data, bring in their expertise, resources and IP, and consequentially leverage synergies and share risk. So far, there are numerous convincing examples demonstrating the potential of PPPs in the pharmaceutical sector [40]. Amongst them, the most prominent are as follows:

- The Biomarker Consortium (source: http://www.biomarkerconsortium.org),
- The Critical Path Institute Consortia (www.c-path.org/consortia.cfm),
- The Innovative Medicine Initiative (www.imi.europe.eu),
- The Serious Adverse Events Consortium (http://www.saeconsortium.org),
- The National Center for Advanced Translational Sciences (https://ncats.nih.gov),
- Bristol–Myers Squibb’s International Immuno-Oncology Network (http://www.immunooncologyhcp.bmsinformation.com),
- The Global Alliance for Vaccines and Immunizations (http://www.gavi.org),
- The Global Fund to Fight AIDS, Tuberculosis and Malaria (http://www.theglobalfund.org/),
- The Stop TB Partnership (http://www.stoptb.org),
- Roll Back Malaria (http://www.rollbackmalaria.org), and
- The FDA initiated the Critical Path Initiative [41].

More specifically, the Innovative Medicines Initiative (IMI) is a PPP established by the European Union (EU) and the European Federation of Pharmaceutical Industries and Associations (EFPIA) [42, 43]. Its goal is to accelerate the development of better and safer medicines. IMI has been engaged in the areas of establishing robust and validated models for drug development, eliminating poorly predictive preclinical models, providing novel biomarkers and drug targets, and establishing more effective tools and methods to predict adverse drug effects [44, 45]. With funding of up to EUR 5 billion, the IMI is stocked to boost the development of new medicines in Europe until 2024. Today, 86 consortia with 593 research teams of EFPIA members, 1214 teams of academic partners and 249 other partner teams are supported by IMI. They focus on all kind of pharmaceutical research topics, including infectious diseases (14%), CNS disorders (10%) or biomarkers (10%) (see Table 2). Sanofi, GSK, J&J and AstraZeneca are the most active contributors from the pharmaceutical industry. They use the potential of IMI intensively by contributing 51, 47, 46, and 44 teams to different projects, respectively (see Table 3). ADAPT-Smart (17 pharma partners), EUPATI (13 pharma partners), GETREAL (13 pharma partners) and PREFER (13 pharma partners) are the projects that have the most EFPIA partner companies. They cover topics of medical research, clinical development (ADAPT-Smart, GETREAL, PREFER) and medical training (EUPATI). To give a few more examples:

**Table 2:** Overview of the IMI projects’ positioning

Topic	Number of projects	Ratio (%)
Biomarkers	9	10
Cancer	4	5
CNS disorders	11	13
Diabetes	3	3
Infectious diseases	12	14
Medical research and clinical development	6	7
NTDs	6	7
Others	17	20
Pain	4	5
Toxicology and drug safety	5	6
Medical training	4	5
Vaccines	5	6
Total	86	

Source: http://www.imi.europa.eu/projects-results/project-factsheets

**Table 3:** Overview of pharmaceutical companies contributing to IMI

	Total number of teams active in IMI projects
Sanofi	51
GlaxoSmithKline	47
Johnson & Johnson	46
AstraZeneca	44
Novartis	36
Pfizer	34
Eli Lilly	32
Roche	31
Bayer	29
Boehringer Ingelheim	28
UCB	25
Novo Nordisk	21
Merck	20
Amgen	16
AbbVie	13
Merck & Co.	10
Takeda	7
BristolMyersSquibb	5

Source: http://www.imi.europa.eu/projects-results/project-factsheets

- The SAFE-T consortium (http://www.imi-safe-t.eu) has been initiated to identify biomarkers for the early detection of drug-related safety issues related to the kidney, liver and vascular systems [46].
- The U-BIOPRED (http://www.europeanlung.org/en/projects-and-research/projects/u-biopred/home) and the PRECISESADS (http://www.precisesads.eu) consortia are both active in the field of personalized medicine in order to address the need for the characterization and classification of severe refractory asthma and autoimmune diseases [47, 48].
- The Open Pharmacological Concepts Triple Store (Open PHACTS, http://www.imi.europa.eu/content/open-phacts) is another PPP with AstraZeneca, Eli Lilly, GSK, Merck, Novartis and Pfizer that complements the 31 consortia members of universities, research organizations, public bodies and governmental organizations. It aims at delivering an open pharmacological space and linking diverse and complementary drug discovery databases to support drug research [49].
- Most likely, the most prominent example of an IMI projects is the European Lead Factory (ELF) that aims at providing new drug targets for all kinds of disease areas [50]. Any scientist from a European academic institution or small- to medium-sized entity (SME) can present a target proposal for High-Throughput-Screening (HTS) or ideas for a compound library. The ELF currently brings together 30 internal partners from private and public organizations, including AstraZeneca, Bayer, and Sanofi (https://www.europeanleadfactory.eu/about/partners/). Starting in 2013, IMI’s goal is to identify high quality small molecule drug candidates. By 2017, the ELF had delivered 300,000 new chemical compounds that were combined with the 300,000 compounds that have been provided by the EFPIA collection to a Joint European Compound Library of more than 500,000 chemical compounds for HTS screening. In addition, by end of 2015, 60 drug target proposals had been evaluated positively and accepted, and more than 500 hits had been handed over to academic institutions and SMEs for further drug development.

The IMI consortia cover topics of high medical need and/or regulatory relevance (see Table 2). The projects generally address topics of the highest scientific and operational complexity that need a multi-stakeholder environment and that do not affect the business success of the participating companies directly. This open innovation model improves companies’ competitiveness and thus may help reduce R&D costs. Meanwhile, it does not increase the competition amongst the companies. It helps reduce the fragmentation of knowledge that is inherent to the IP-driven pharma industry. Other projects of IMI also stimulate the interest of pharmaceutical companies in research topics that would not have been examined without rewards, such as antimicrobials (see ENABLE) or Ebola vaccines (see EBOVAC1).

In summary, pharmaceutical companies use PPPs as a tool of precompetitive collaboration with peers:

- To increase scale,
- To strengthen research networks,
- To share risk with industry partners,
- To integrate external (university) know-how,
- To access public financial resources,
- To accelerate the exploitation of innovation,
- To serve society and increase reputation, and
- To improve R&D productivity [51].

In addition, they need to meet the following challenges to leverage the potential of PPPs:

- Pool and share the right data that are relevant for the PPPs,
- Manage the alliances professionally,
- Exclude the IP relevant (competitive) topics,
- Manage the IP rights if such rights result from a PPP,
- Manage the cultural conflicts of partners, and
- Align the diverse interests of partners that are coming from academia and industry.

## Pharma innovation centres and research alliances to leverage synergies from open innovation

Another path to open up R&D and to access external innovation is pharma innovation centres. They may be best described as hybrid models of centralized R&D mixed with elements of open innovation. Some of the biggest players in the pharmaceutical sector have embraced this new concept that brings scientist from pharmaceutical companies and experts from the academia together to solve problems, provide new solutions and deliver innovative products.

- Pfizer started in 2010 their Global Centers for Therapeutic Innovation (CTIs), a USD 85 million partnership with the University of California at San Francisco (UCSF). This one example out of eight planned CTIs shall become part of Pfizer’s BioTherapeutics Research Group and it is Pfizer’s way to leverage synergies from open innovation at the interface of translational research between Pfizer and academic medical centres (http://www.pfizercti.com). The idea is that Pfizer adds its developmental knowledge, financial resources and human resources to the collaboration. Meanwhile, it expects to benefit from the research expertise in disease areas, target biology and patient populations from their academic partners. Partners in the CTI network include 25 academic institutions, four patient foundations, the National Institute of Health (NIH) and large hospitals such as the Medical Center at Columbia University, the Tufts Medical Center or Mount Sinai Hospital. Together with the NIH, Pfizer plans to identify new drugs that serve both partners’ strategic interests. NIH scientists provide more of the research aspects in the collaboration (such as disease-related pathways or mechanisms as potential drug targets), while Pfizer enables the NIH to move new drug projects into therapeutic development using industry-standard translational tools and Pfizer’s developmental expertise.

Other large pharma companies have also started their own innovation centres:

- Bayer HealthCare, the German Cancer Research Center (DKFZ) and the National Center for Tumor Diseases (NCT) initiated a drug discovery alliance in 2009 where DKFZ and NCT provide their expertise in cancer research and tumour biology, and Bayer brings in its know-how in drug R&D. The goal of this strategic partnership is technology transfer from the DKFZ to Bayer [52].
- Bayer HealthCare recently opened a centre in San Francisco to identify and manage partnerships with academic and biotech researchers in the USA [53].
- The German company Merck established a new innovation centre located at their headquarters to open Merck’s R&D towards innovation from the outside and to provide a platform for external innovation to translate their ideas into real business cases [54].
- J&J started its Life Science Innovation centre in San Diego with a Concept Lab that provides a platform for start-up companies in the earliest phase and with the Open Collaboration Space that gives the start-up companies desk space later in their development. With both programmes, J&J enables innovators to get an early proof-of-concept for their ideas by offering infrastructure, equipment and resources [55].
- GSK experimented much earlier in the field of open innovation with the Center of Excellence for External Drug Discovery (CEEDD). At its start in 2005, CEEDD’s goal was to better access external technologies using a small team of approximately 20 GSK scientists to work across all therapeutic areas and facilitate partnerships from drug discovery to PoC. Until its closure in 2012, CEEDD managed a total of 16 partnerships. One example for collaboration under this umbrella is the GSK alliance with ChemoCentryx, which was established in 2011 to access the technology of chemokine-based therapeutics. After one of the trials failed and the chemokine receptor 9-inhibitor did not achieve its primary endpoint for the treatment of Crohn’s disease, the programme was discontinued. Despite such drawbacks, CEEDD was successful in establishing both the internal and the external paths to access drug candidates at GSK and it helped to fill GSK’s early stage pipeline currently covers nearly half of its projects that are externally sourced [56].
- Since more than a decade Novartis and the MIT are collaborating on continuous manufacturing (https://novartis-mit.mit.edu/). In 2018 they added a partnership with MIT’s Computer Science and Artificial Intelligence Laboratory (CSAIL) on patient real-time motion monitoring [73].
- Novartis has also successfully collaborated with selected institutions such as the Dana–Farber Cancer Institute on cancer alliances or the University of Pennsylvania on the chimeric antigen receptor (CAR) T cell [57]. In a recent interview, Novartis Institutes for BioMedical Research’s (NIBR) president Jay Bradner announced a division-wide programme to enhance the external focus and foster external collaborations in several ways [58]. A faculty of scholars will be invited to join NIBR to lead their own projects that “*would not be accessible in their home institutions*” [58]. This could be access to technologies but also libraries of molecules to test new concepts. The programme is special and different from other open innovation models since it focuses on sourcing new ideas into a company and provides leading academic scientists and innovators the opportunity to benefit from pharma’s strengths. Thus, this turns the effort into a collaborative partnership to the benefit of science and patients.
- In addition Novartis has started “Genesis labs” to offer employees the opportunity to suggest high-risk, high-reward transformational projects and apply for resources, funding and lab space.
- Another example of a pharma innovation centre is Takeda’s Center for IPS Cell Research Application (CiRA) at Kyoto University (TCiRA). Launched in 2015, its goal is to investigate cell therapies for the treatment of cardiovascular, metabolic, neural and cancer diseases. While Takeda provides 10 years of funding of approximately US$ 177 million, research management expertise, Takeda researchers, access to its compound library and access to the research facilities at its Shonan Research Center in Japan, it can benefit from the world-class science of the CiRA team that is headed by director Shinya Yamanaka, the 2012 Nobel Prize winner on induced pluripotent stem (iPS) cells.
- Takeda is one of the global players that has reduced its internal R&D over the past years and that increasingly uses the potential of external R&D [59, 60]. It recently announced a collaboration with the Tri-Institutional Therapeutics Discovery Institute (TDI) in New York, which is a consortium of three institutions (Cornell University, Rockefeller University, and Memorial Sloan Kettering Cancer Center), to support target research [61]. It also collaborates with J&J and OrbiMed Advisors LLC by co-investing in the Israeli biotech accelerator FutuRx with the purpose to fund medical breakthrough innovations that can be spun off in the form of new, independent companies [62].

These examples demonstrate how access to world-class science is facilitated by agreeing on a long-term partnership with academic institutions. The further advantages of innovation centres are:

- They bypass tedious long-lasting licensee-licensor negotiations in subsequent drug licensing;
- They allow access to internal scientific resources within the remit of the arrangement, which is an important flexibility given the risks of pharmaceutical R&D;
- They allow R&D to familiarize themselves with new technologies or therapeutic indications without the need to make significant investments upfront; and  
- They give access to potential new drug candidates.

Conversely, the academic and biotechnology partners also benefit from such collaborations. They get access to the drug discovery, developmental expertise and financial resources of pharmaceutical companies without the necessity to seek a strategic investor and without the risk of losing control of their own drug candidates. Academic institutions such as UCSF, Harvard University or the University of Pennsylvania have realized the potential of long-term research alliances and have all formed partnerships with major research-based pharmaceutical companies such as Bayer, Sanofi, Boehringer Ingelheim or Novartis.

It is important to state that any kind of collaboration, alliance or partnership also creates a set of specific challenges. Among the latter are increased management complexity, coordination costs and the risk of IP failure. Academic research is often early-stage with respect to commercialization, and substantial additional work and financial investments are needed before a return-on-investment can be expected. There is the constant challenge of early result publication since academia expects and needs findings to be published, while companies need to protect their assets via IP. These conflicts are not easy to solve and require tactful manoeuvring and mutual understanding of the different priorities.

## Virtualization of R&D to increase efficiency

Virtual R&D pharmaceutical companies look to control overhead costs by operating a critical mass of internal expertise and complementing this with external know how and resources to drive internal R&D. In general, the fixed costs of R&D are reduced through the transfer of less intensively utilized services to partner companies, economies of scale and scope are leveraged, and flexibility is increased by choosing the best R&D service providers globally [62–64]. This dynamic has four major advantages:

- A reduction of internal complexity due to concentration,
- A focus of resources on core technologies that may provide a competitive edge,
- A mitigation of investment risks to partner companies, and
- A reduction of external interfaces, since the task is now shared with the partner companies.

So far, virtual R&D has been successfully applied predominantly by SMEs, such as Fulcrum, Shire, Debiopharm or Endo Pharmaceuticals. Eli Lilly is an example of a global pharma company that also uses this open innovation model. It realized that the big pharma franchise approach of a central R&D may not be applicable for all R&D projects. Thus, it established the Chorus group as a lean and efficient way to PoC that was better suited to drug candidates that rely on targets with a lower target validation status and higher inherent technical risks. Chorus is a small and independent entity that has the goal to run clinical studies with a minimum required data package in a “quick win, fast fail” model. It aims at advancing projects from candidate selection to PoC and to provide the candidates for Eli Lilly’s phase III pipeline [65]. It has the science-driven business model of a biotechnology company with flat hierarchies, fast decision-making processes and a project organization as follows:

- Projects have dedicated project-budgets,
- The focus is on phase I and clinical PoC studies,
- Projects are managed with small expert teams, and
- The main tasks are run by intensive outsourcing.

By 2016, Chorus managed a portfolio of 15 drug projects with clinical trials in 19 countries with 40 full-time equivalent staff. Chorus was able to run its operation with 25% of its budget as fixed overhead costs and 75% of the financial resources allocated to the external costs of the drug projects [65]. Overall, Eli Lilly’s concept of virtual R&D met its proof-of-principle since its average success rate in phase II improved significantly (54% Chorus vs. 29% traditional Eli Lilly) and its productivity increased by a factor of 3–10 compared with Eli Lilly’s traditional clinical development model [65].

Debiopharm is an additional example of the success of virtual R&D. It has been operating its business model successfully over the past 39 years by utilizing its internal expertise and absorptive capacities (60% of staff hold a Ph.D. or M.D.) in drug development to identify and in-license promising therapies and develop them with the aim to outsource it to a partner with sales and marketing expertise. The company operates an open innovation model that engages external experts and service providers for project-related activities, with the flexibility to include vendors with the latest technologies and solutions to the question at hand. In consequence, Debiopharm has one of the highest revenue rates earned per employee across pharmaceutical companies, indicating the advantage of this open innovation model (see Table 4).

**Table 4:** The comparison of average revenue earned per employee across different pharmaceutical companies

Company	Type	Founded	Number of employees	Average 10 years (2006–2016) revenue (M$)	Revenue per employee ($)
Helsinn	NRDO	1976	680	320	470,588
Jazz Pharma	NRDO	2003	1040	615	591,346
Tesaro^+^	NRDO	2010	446	0.45	1010
Puma Biotech^++^	NRDO	2010	183	0	0
Clovis	NRDO	2009	278	0.14	504
Vanda Pharma	NRDO	2002	148	38	256,756
Debiopharm	NRDO	1979	150	345	2,300,000
Astra Zeneca	Large Pharma	1999	59,700	23,000	385,260
Novartis	Large Pharma	1996	12,3000	47,000	382,114
Sanofi	Large Pharma	2004	110,000	32,500	295,454
Eli Lilly	Large Pharma	1876	42,000	21,500	511,905
Bayer	Large Pharma	1863	115,000	43,000	373,913
Pfizer	Large Pharma	1849	96,500	51,000	528,497
GSK	Large Pharma	2000	99,300	26,400	345,619
Amgen	Large Biotech	1980	19,200	20,000	1,041,667

*NRDO* ‘No Research, Development Only’ companies that do not conduct early research and have not chosen to conduct sell and market their own assets; ^+^ calculated over 6 years; ^++^ calculated over 7 years

One of the core elements of the open innovator type “knowledge leverager” is the virtualization of R&D [4]. Shire is one of the prototypes of this model. Over the last 30 years, they have established themselves as one of the world’s fastest growing specialty pharma companies. Currently, they are one of the major players in rare diseases with a market capitalization of approximately USD 40 billion. Shire does not conduct any early internal research but chose to fill its portfolio through the acquisition of companies with late phase assets that must be driven through the developmental process for approval and commercialization. Shire is a lean organization that is extremely cost effective, thus reflecting their high EBIT-margins, annual growth and flexibility with respect to changing environments and opportunities. Shire focuses its internal efforts around efficient clinical development, fostering relationships with key opinion leaders and advocacy/patient groups around diseases of interest, and preparing the market (specifically around pricing and reimbursement).

Overall, the common denominator of these examples is a project-oriented organization of scientific and industry experts with excellent networks with industrial partners and academic institutions, a limited number of permanent and costly headcounts and infrastructures, flexible access to external resources and knowledge on demand and an organizational structure that enables fast decision-making (Table 5).

**Table 5:** Success factors of virtual R&D

Advantages of a virtual R&D organization	Essential elements to operate a virtual R&D organization
Instant access to new technologies and external resources on demand	Excellent industry and academic networks
Reduced capital requirements (overhead and infrastructure)	Professional, project, portfolio and alliance management skills
Simple governance structure	Simple governance structure
Reduced bureaucracy and faster decision making	Collaborative scientific and medical support from those who understand drug development
Flexibility in selecting optimal services providers	Excellent expertise in risk management and financial valuation
Mitigated financial risk	Outperforming licensing and acquisition strategies and skills
Reduced time to market	Admirable outsourcing skills
	Excellent communication and motivation skills to manage external project teams

## Why is the potential of open innovation not fully utilized in the pharmaceutical industry yet?

During the past years, the industry has experimented with some collaborative R&D concepts that may help companies to open up their R&D towards external knowledge and technologies. Pharma innovation centres, research alliances, and the seeker-like approach in active crowdsourcing are all open innovation concepts that are new for the industry, but that fit more with the traditional, predominantly internal R&D models of the big pharma companies. The industry also tested newer open innovation models, such as PPPs or open source, but primarily did so in research areas that do not affect its major franchises. Only Eli Lilly, Bayer Healthcare and some smaller players progressed in implementing more open concepts, which is surprising in light of the enormous challenges that the industry is facing. Therefore, why has the industry not yet used the full potential of open innovation?

For example, implementing active crowdsourcing and running a proprietary crowdsourcing platform is related with challenges and requirements, such as

- Company’s R&D processes need to be extroverted to implement external solutions,
- R&D employees need to be open to ideas coming from the outside,
- The R&D organization needs to have sufficient resources to access external innovation,
- Internal champions need to be able to take the lead on external proposals,
- An environment needs to exist that enables bilateral agreements,
- Concepts need to be in place regarding how to financially reimburse solvers properly and
- Clear IP regulations need to be implemented that consider the needs of both parties.

In short, using the full potential of open innovation would be linked to a change of the complete R&D model. Possible reasons for not completely embracing the full potential of open innovation could be related to the factors of (1) legacy, (2) risk, and (3) control.

### Legacy

1. Changing cultures: Shifting an R&D organization’s traditional R&D model to a new one could be viewed as an unnecessary attempt since the old R&D strategy is often hard-wired in the DNA of the organization and the culture of its people.
2. Changing people: The typical pharma scientist in the big pharma franchise model still tends to be a more introverted specialist, whereas working in an environment of external collaboration or virtual R&D requires individuals to be drug developers with excellent communicative and collaborative skills and networks that they bring to the project teams.

### Risk

1. Risk associated with academic research: Drug development is certainly more costly and challenging today than in the past. The expectation to deliver differentiated products in stratified populations in existing markets increases the bar for success. There is no doubt that academic institutions and biotech groups are conducting innovative research in certain disease areas and generating new targets in new indications. However, a great deal of these targets are not clinically validated.
2. Return-on-investment risk of licensing: Recent deals seem to suggest that for relatively early projects, the market is willing to pay several multiples of the value of an asset, thus making the internal generation of assets more attractive again.
3. Financial risk of restructuring: The costs for restructuring a global R&D group located at different sites to become a slim virtual organization and the effort involved in changing the mindset and culture would be substantial.

### Control

1. Controlling IP: External innovation and IP often tend to result in multiple contributors where each requires a percentage of the royalties and an unrealistic upfront payments and milestones.
2. Controlling markets and competitors: Although big pharma has moved to a leaner and more focused R&D model for non-core assets, they have been reluctant to apply a similar model to their core assets. These major franchises have been established over the years in specific therapeutic areas where a large amount of investments has been realized in infrastructure, established knowledge and expertise, which they are reluctant to dismantle since it provides a competitive edge.

For example, the difficulties associated with the change of the R&D model and culture can in retrospect be exemplified by the ALTANA-Nycomed–Takeda mergers. In 2006, the Danish Nycomed AS bought the German ALTANA AG. Nycomed came along with a team of approximately 100 full time equivalents who have been working according to the “knowledge integrator” approach, whereas ALTANA’s tenfold larger R&D organization traditionally had been operating according to the “knowledge creator” model. Nycomed’s intention was to run the newly generated joint R&D organization according to the open innovator of a “knowledge leverager” and therefore make the organization more extroverted than ALTANA traditionally was. Although there were many isolated project successes, the attempt to shift the R&D model and employ more open innovation concepts was ultimately not embraced on a broad basis and was eventually abandoned when Nycomed was sold to Takeda in 2012. The goal to establish a “knowledge leverager” model was driven by the head of R&D and the next two management levels, but it was never fully supported by other C-suite leaders whose focuses were to defend their organizations, structures and approaches. The more traditional knowledge creator model of ALTANA acted as a brake for the execution of the new open innovator by focusing too intensively on internal innovation and maximizing the value of late-stage assets. The predominant organizational R&D culture also limited the influence of the open innovation strategy implementation. R&D employees were often distrustful of any replacement of internal capacities by external resources, in-house scientists were sceptical of any external asset to be evaluated for licensing (“not-invented-here”) and R&D managers were too anchored in the competitive mode to understand how to build a culture of collaboration and creative working with multiple partners and stakeholders.

To conclude, the influence of the more traditional organizational culture should have been reduced by:

- Challenging the automatic distrust towards external partners who are motivated differently,
- Questioning the over-identification with internal capabilities and thereby overcoming the “not-invented-here syndrome”,
- Letting go of the limiting competitive reactiveness that sees everyone outside of the organization as a potential threat, and
- Reducing the influence of control in decision making.

Changing an R&D model involves cultural change. Thus, this change takes time and is a huge effort. A McKinsey survey of 3199 executives around the world found that only one out of every three transformations succeeds [66]. This low success rate prompts the question of how pharma can increase its chances of success, for example, when changing its R&D model to the open innovator of a “knowledge leverager” and copy the successful biotech business model that continuously outperformed large pharma with respect to their R&D output [67]. Possible answers include:

- Incubate a new project that is geographically and structurally separate and protect it from the rest of the organization. If the project grows and succeeds, gradually redirect investment funds towards it and away from traditional operations.
- Recruit an elite team from within the old organization and from outside along with the downsizing or closure of the existing operations.
- Establish a company-based R&D-independent fund that supports new innovative ideas from employees. These can be projects that either do not have a home in a functional line or therapeutic area or that are (currently) regarded as out of scope.

In any case, any new concept to open up pharma R&D needs to be linked to a new and more entrepreneurial organizational culture and an open and collaborative mindset of the people. The challenge for the more conservative large pharma companies in general is to maintain the entrepreneurial spirit at a large scale, which must also involve innovative approaches to legacy, risk and control. Some large pharma (e.g., Eli Lilly, GSK or J&J) have experimented with smaller, more autonomous units, or even new R&D models (e.g., Nycomed), but may have ignored the underlying influence of the old R&D model, thus leading to mixed results. Eli Lilly has been very successful with Chorus. G4T is another positive example of how an open innovation model can be implemented. In addition, Actelion can also be seen as a successful example and representative since innovation and entrepreneurial spirit is their motor, as they continuously kept investing in its R&D, even in case of drawbacks. The fact that even after having sold Actelion to J&J for USD 30 billion the “Idorsia” (the R&D part of Actelion) was not part of the deal (and will remain operational and independent) underscores that R&D can be both very profitable and successful if well managed [68].

## What can big pharma learn from the new open innovator types?

Companies following a more traditional R&D Model (such as the open innovator “knowledge creator”) have a fundamentally different view of R&D than those companies that operate a completely open R&D concept. The open innovator of a “knowledge creator” is based on a predominantly internally generated R&D pipeline with a preference for introverted innovation management [4] that is based on highly innovative research approaches, the assessment of medical needs (to address real needs) and the understanding of basic biological principles (work on the right targets and thus reduce technical risks). In contrast, companies with a virtual R&D model are innovative in their business models (i.e., with respect to scouting, business development and licensing activities) rather than in their research approaches.

Therefore, what can the more traditional big pharma companies learn from this more open R&D model?

1. Expand the portfolio strategy: Re-position projects that would have been put on hold or out-licensed. Since the true commercial value of an asset is often difficult to assess, decisions should be made based on their transformative potential rather than sales predictions.
2. Reduce costs: Big pharma companies need to focus more on project-related expenditures rather than on capacity or capability building. With today’s rapidly changing environment, they often can be insourced and might be outdated once they are built internally.
3. Increase flexibility: This relates to a mindset that implies that a discovery that might not fit the corporate goals or project portfolio still can be of high value or impact to another part of the organization or therapeutic area. Assumed or perceived R&D strategies with seemingly defined out-of-scope areas can limit employees’ creativity and ownership. Thus, considering opportunities should become a part of big pharma’s mindset.

## Conclusions

Currently, pharmaceutical companies can benefit from numerous sources of external innovation, including open sourcing, crowdsourcing, public private partnerships, dedicated innovation centres and research alliances. To this end, pharmaceutical companies need to develop absorptive capacities where external knowledge gets identified, valuated and actively incorporated. To release the next wave of innovation in the pharmaceutical industry, pharma companies need to tap external sources of innovation. However, first they need to be innovative in the way that they design new organizations that are fit for external innovation. Depending on the companies’ sizes, their portfolio breadths and their strategic goals, companies need to apply different open innovation models ranging from simply adding elements of open innovation to their existing R&D models to completely virtualizing their R&D. The available options are pretty different from each other and have specific advantages and shortcomings. A best-in-class or ones-size-fits-all approach cannot be recommended currently. Instead, drug innovators need to choose the option that best fits their current needs and strategic directions. Companies aiming at leveraging the full potential of open innovation first need to adapt their approaches with respect to the three success factors, namely, legacy, risk and control. Second, they need to fully engage their external partners and resources in a truly collaborative model, which provides benefits to all partners involved.

## Abbreviations

ANDI: African Network for Drugs and Diagnostics Innovation
BMS: Bristol–Meyers Squibb
CAR: chimeric antigen receptor
CEEDD: Center of Excellence for External Drug Discovery
CiRA: Center for iPS Cell Research Application
CNS: Central Nervous System
CTIs: Centers for Therapeutic Innovation
DNDi: Drugs for Neglected Diseases Initiative
DKFZ: German Cancer Research Center
EBIT: earnings before interest and taxes
EFPIA: European Federation of Pharmaceutical Industries and Associations
ELF: European Lead Factory
EU: European Union
FDA: Food and Drug Administration
GSK: GlaxoSmithKline
G4T: Grants4Targets
HTS: High-Throughput-Screening
IMI: Innovative Medicines Initiative
IP: intellectual property
iPS: induced pluripotent stem
J&J: Johnson & Johnson
MIT: Massachusetts Institute of Technology
MMV: Medicines for Malaria Ventures
NCT: National Center for Tumor Diseases
NIBR: Novartis Institutes for BioMedical Research’s
NIH: National Institute of Health
OS: open source
OSDD: open source drug discovery
PoC: proof-of-concept
PPP: public–private partnerships
R&D: research and development
SMEs: small and medium-sized enterprises
TB Alliance: Global Alliance for Tuberculosis Drug Development
TCiRA: Takeda CiRA Joint Program for iPS Cell Applications
TDI: Tri-Institutional Therapeutics Discovery Institute
TDR: Special Program for Research and Training in Tropical Diseases
TPP: target product profile
UCSF: University of California at San Francisco
UNECA: United Nations Economic Commission for Africa
WHO: World Health Organization
